# Integrative genomics reveals hypoxia inducible genes that are associated with a poor prognosis in neuroblastoma patients

**DOI:** 10.18632/oncotarget.12713

**Published:** 2016-10-17

**Authors:** Mark A. Applebaum, Aashish R. Jha, Clara Kao, Kyle M. Hernandez, Gillian DeWane, Helen R. Salwen, Alexandre Chlenski, Marija Dobratic, Christopher J. Mariani, Lucy A. Godley, Nanduri Prabhakar, Kevin White, Barbara E. Stranger, Susan L. Cohn

**Affiliations:** ^1^ Departments of Pediatrics, University of Chicago, Chicago, Illinois, 60637, United States of America; ^2^ Committee on Clinical Pharmacology and Pharmacogenomics, University of Chicago, Chicago, Illinois, 60637, United States of America; ^3^ Institute for Genomics and Systems Biology, University of Chicago, Chicago, Illinois, 60637, United States of America; ^4^ Department of Human Genetics, University of Chicago, Chicago, Illinois, 60637, United States of America; ^5^ Center for Research Informatics, University of Chicago, Chicago, Illinois, 60637, United States of America; ^6^ Department of Medicine, University of Chicago, Chicago, Illinois, 60637, United States of America; ^7^ Center for Data Intensive Science, University of Chicago, Chicago, Illinois, 60637, United States of America

**Keywords:** neuroblastoma, hypoxia, metabolism, RNA expression, pathway analysis

## Abstract

Neuroblastoma is notable for its broad spectrum of clinical behavior ranging from spontaneous regression to rapidly progressive disease. Hypoxia is well known to confer a more aggressive phenotype in neuroblastoma. We analyzed transcriptome data from diagnostic neuroblastoma tumors and hypoxic neuroblastoma cell lines to identify genes whose expression levels correlate with poor patient outcome and are involved in the hypoxia response. By integrating a diverse set of transcriptome datasets, including those from neuroblastoma patients and neuroblastoma derived cell lines, we identified nine genes (*SLCO4A1, ENO1, HK2, PGK1, MTFP1, HILPDA, VKORC1, TPI1, and HIST1H1C*) that are up-regulated in hypoxia and whose expression levels are correlated with poor patient outcome in three independent neuroblastoma cohorts. Analysis of 5-hydroxymethylcytosine and ENCODE data indicate that at least five of these nine genes have an increase in 5-hydroxymethylcytosine and a more open chromatin structure in hypoxia versus normoxia and are putative targets of hypoxia inducible factor (HIF) as they contain HIF binding sites in their regulatory regions. Four of these genes are key components of the glycolytic pathway and another three are directly involved in cellular metabolism. We experimentally validated our computational findings demonstrating that seven of the nine genes are significantly up-regulated in response to hypoxia in the four neuroblastoma cell lines tested. This compact and robustly validated group of genes, is associated with the hypoxia response in aggressive neuroblastoma and may represent a novel target for biomarker and therapeutic development.

## INTRODUCTION

It is well recognized that more effective therapy is needed for children with high-risk neuroblastoma [1]. Although modern, multi-modality treatment strategies have led to improved outcome for these patients [2], fewer than half are cured. Further, current risk stratification criteria cannot distinguish those high-risk patients who will achieve long-term survival with standard treatment approaches from those who will relapse or develop progressive disease. Thus, an improved understanding of the molecular mechanisms that contribute to the clinically aggressive neuroblastoma phenotype is critical for refining risk stratification and the development of novel therapeutics.

Aggressive solid tumors such as high-risk neuroblastoma, are known to contain regions of severe hypoxia, subsequently affecting numerous cellular pathways [3]. Hypoxia induces the stabilization of Hypoxia Inducible Factor (HIF), leading to activation of downstream targets in metabolism, angiogenesis, and cell division [4]. Neuroblastoma cell lines grown under hypoxic conditions have an undifferentiated, aggressive phenotype and altered gene expression in HIF-regulated genes [5]. In addition, hypoxia has been shown to increase the metastatic potential of neuroblastoma cells [6], enhance stem cell-like phenotype [7], and alter proliferation [8]. Activation of the hypoxia response pathways may have prognostic implications for survival in patients with neuroblastoma [9], although which aspects of the hypoxia response are driving the biology of aggressive neuroblastoma tumors remains unclear.

Collaborative initiatives such as The Cancer Genome Atlas (TCGA) have been able to aggregate clinically annotated multidimensional tumor genomics data from large numbers of adult patients [10]. Efforts to achieve similar results through the Therapeutically Applicable Research to Generate Effective Treatments (TARGET) pediatric initiative remain difficult due to relative rarity of many pediatric cancers. In this study, we were able to utilize publically available transcriptome data from multiple cohorts of clinically annotated neuroblastoma tumors, together with transcriptome data from hypoxia-treated neuroblastoma cell lines to identify genes with expression levels associated with both poor patient outcome and an aggressive hypoxia regulated phenotype. Using differential expression, 5-hydroxymethylcytosine quantification, Kaplan-Meier and Cox regression methods, we identified nine hypoxia-related candidate genes that play a role in neuroblastoma pathogenesis. These genes were experimentally validated to have increased expression in response to hypoxia in multiple neuroblastoma cell lines *in vitro* and may also impact the ability of neuroblastoma cells to thrive in hypoxia.

## RESULTS

### Genes associated with outcome in neuroblastoma

**Figure 1:**
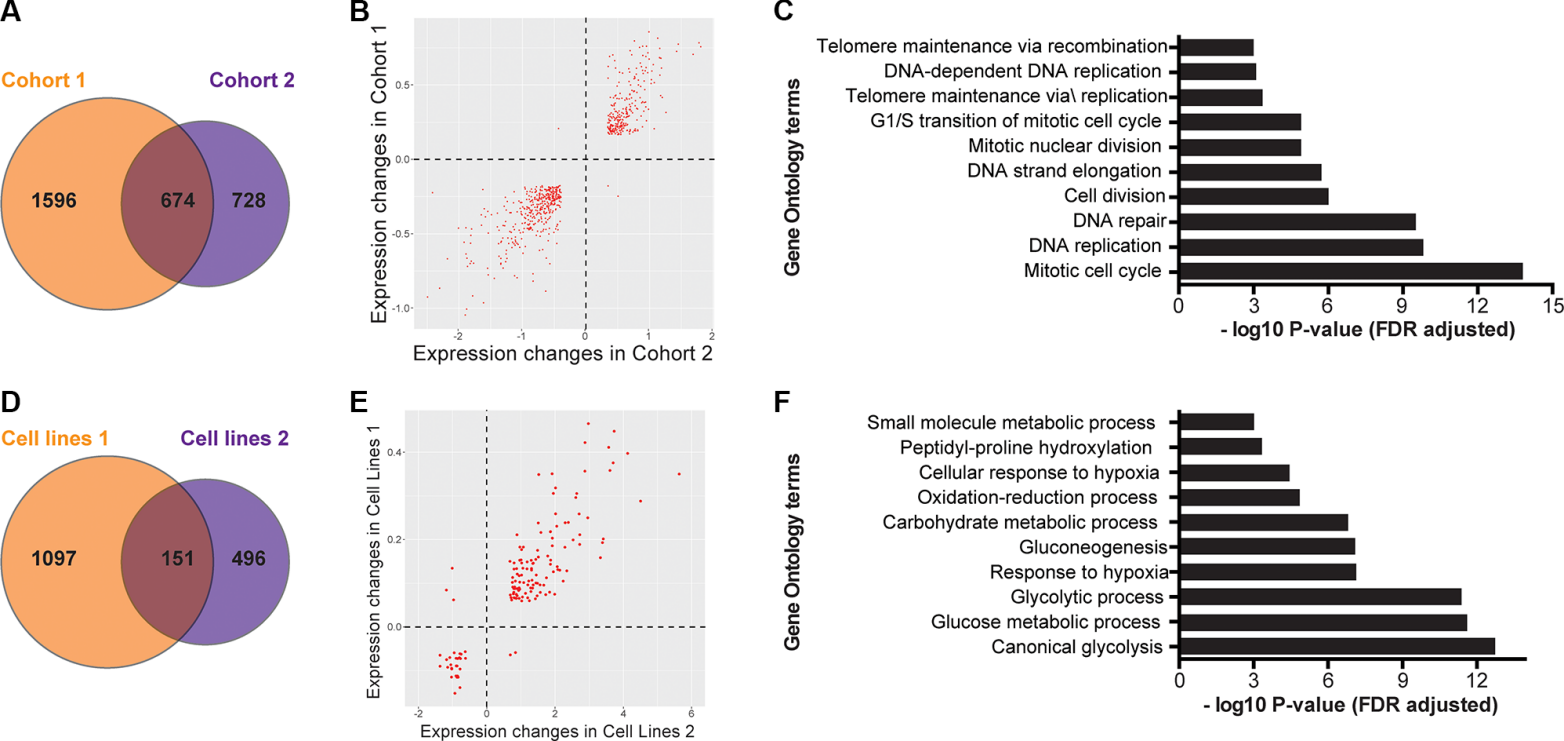
Differentially expressed gene sets show commonalities in both patient and cell line cohorts (**A**) Unique and shared genes between patient cohorts 1 and 2. (**B**) The direction of expression change between common DEGs in survivors and deceased patients is consistent between cohorts for all but five genes. (**C**) Gene Ontology analysis of differentially expressed genes in both patient cohorts. (**D**) Unique and shared genes between cell line experiments. (**E**) The direction of expression change between common DEGs in normoxic and hypoxic cell lines is consistent between cohorts for all but five genes. (**F**) Gene Ontology analysis of differentially expressed genes in both cell line datasets.

We initially sought to define a set of genes whose transcription levels are correlated with survival in patients with neuroblastoma, as they may play a biological role in tumor growth, with the potential to be clinically actionable. To do this, we first analyzed microarray expression data from 478 diagnostic neuroblastoma tumors (Cohort 1: EMBL accession E-MTAB-179) [11]. The tumor samples are clinically annotated, and the cohort includes stage 1 (*n* = 119), stage 2 (*n* = 80), stage 3 (*n* = 69), stage 4 (*n* = 148), and stage 4S (*n* = 62) neuroblastoma patients. Ninety-one of the patients died, and 74 patients had *MYCN*-amplified tumors. Using a linear regression model comparing patients who survived to those who did not (see Methods), we identified 6664 differentially expressed genes (DEGs) with a False Discovery Rate of less than 5% (FDR < 0.05) after controlling for *MYCN*-amplification. After restricting this set to only those within the top 10% of fold change and a FDR less than 1% (FDR < 0.01), 2270 genes remained (Figure 1A). A Gene Ontology GO analysis [12] revealed that top biological processes that were significantly enriched for the 1169 up-regulated DEGs in deceased patients were associated with regulation of cell cycle and mitosis (Supplementary Table S2), while there was no significant pathway enrichment for the 1101 down-regulated DEGs.

In order to validate these findings, we analyzed transcriptome data from a second and independent cohort (Cohort 2: GEO accession GSE16254) containing 88 patients with neuroblastoma [13]. This cohort included patients with stage 1 (*n* = 8), stage 2 (*n* = 15), stage 3 (*n* = 13), stage 4 (*n* = 40), and stage 4S (*n* = 12) tumors. Thirty-three of the patients died, and 16 patients had *MYCN*-amplified tumors. Using identical linear models and criteria for fold change and FDR, we identified 1402 DEGs in this validation cohort (Figure 1A). In this analysis, the 591 up-regulated DEGs in deceased patients were also enriched for genes involved in cell cycle processes (Supplementary Table S3), and again there was no pathway enrichment for the 811 down-regulated DEGs.

A total of 674 genes were differentially expressed in both patient cohorts (Figure 1A), nearly all (671/674) with consistent changes in directionality of expression between survivors and deceased patients in both cohorts (Figure 1B). A permutation test showed that this degree of overlap of genes between the two cohorts is significantly greater than that expected by random chance (*P* < 1 × 10^−6^, Supplementary Figure S1A). Similar to the DEG of each individual patient cohort, the top GO terms for the 271 overlapping up-regulated DEG from both cohorts were related to cell cycle and mitosis (Figure 1C, Supplementary Table S4) while there were no significantly enriched GO terms in the group of 400 overlapping down-regulated DEGs. Our findings are consistent with prior reports as cell cycle genes are well established as being up-regulated in more aggressive neuroblastoma [14, 15] and the number of dividing cells seen by pathologic examination has long been an indicator of higher-risk disease and poor outcome in patients [1]. However, it is impractical to determine which of these 271 genes are functionally most important for accelerating neuroblastoma cells through the cell cycle. Thus, we sought to identify a subset of these genes that are correlated not just with patient outcome, but also with the hypoxia response itself, as it has been previously shown that hypoxia increases the aggressive phenotype of neuroblastoma and affects progression through the cell cycle [16, 17].

### Gene expression patterns from neuroblastoma cells grown in hypoxia

In order to identify genes that are hypoxia-regulated in neuroblastoma, we analyzed two independent transcriptome datasets of neuroblastoma cell lines that were exposed to laboratory hypoxia. In the first dataset, SK-N-BE2 cells were grown in 1% oxygen and compared to those that were grown in normoxia [18]. RNA-seq analyses of expression differences between these groups identified 1248 DEGs (FDR < 1% and top or bottom 10% log2 fold change, Figure 1D). The 753 up-regulated DEGs were enriched for several biological processes, including the NCI HIF-1α transcription factor network (*P* = 3.8 × 10^−16^), and the GO terms canonical glycolysis (*P* 3.6 × 10^−14^), and synthesis of cholesterol (*P* = 6.7 × 10^−14^; Supplementary Table S5) while the 496 down-regulated genes had no significantly enriched terms [12].

To validate these findings further, we analyzed an independent dataset in which 11 neuroblastoma cell lines were grown in 21% or 1% O_2_ [9]. From this dataset, we identified 647 DEGs between normoxia and hypoxia (Figure 1D). Consistent with our findings using the RNA-seq data from the SK-N-BE2 cell line, we also found that 324 up-regulated DEGs in this dataset were also enriched for genes in HIF-1α transcription factor network (*P* = 1.6 × 10^−10^), canonical glycolysis (*P* = 5.4×10^−9^) and glucose metabolism (*P* = 8.8×10^−9^; Supplementary Table S6), while the 323 down regulated genes demonstrated no consistently enriched processes. A total of 151 genes were regulated by hypoxia in both datasets (Figure 1D), and a permutation test revealed such an overlap between the two datasets was highly significant (*P* < 1 × 10^−6^; Supplementary Figure S1B). Furthermore, all but five of 151 DEGs showed consistent directionality of expression change from normoxia to hypoxia in both datasets (Figure 1E) and GO analysis of these 146 hypoxia regulated genes also showed the 119 consistently up-regulated genes were highly enriched for metabolic and hypoxia related processes (Figure 1F, Supplementary Table S7).

**Figure 2:**
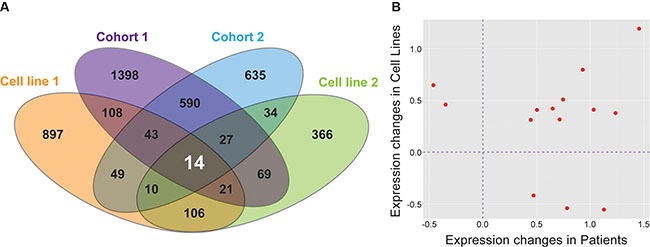
Nine genes are differentially expressed in all datasets and are significant on multiple analyses (**A**) Unique and shared genes between patient Cohort 1, patient Cohort 2, and both cell line experiments. (**B**) The direction of expression change between common DEGs in all datasets is consistent for nine genes.

Next we compared the 151 hypoxia regulated genes identified from neuroblastoma cell lines exposed to hypoxia to the 841 DEG associated with survival in the patient cohorts and identified 14 genes common to all four datasets (Figure 2A). Among these 14 genes, nine (*SLCO4A1*, *ENO1*, *HK2*, *PGK1*, *MTFP1*, *HILPDA*, *VKORC1*, *TPI1*, and *HIST1H1C*) showed consistent directionality of expression changes in both cell line experiments and patient cohorts (Figure 2B), suggesting a similar effect in both cell lines and aggressive tumors. A permutation test revealed that the degree of overlap, nine shared genes in all four datasets differs significantly from random expectation (*P* = 8 × 10^−5^; Supplementary Figure S2). We considered these nine genes as genes of interest for further analyses.

**Figure 3:**
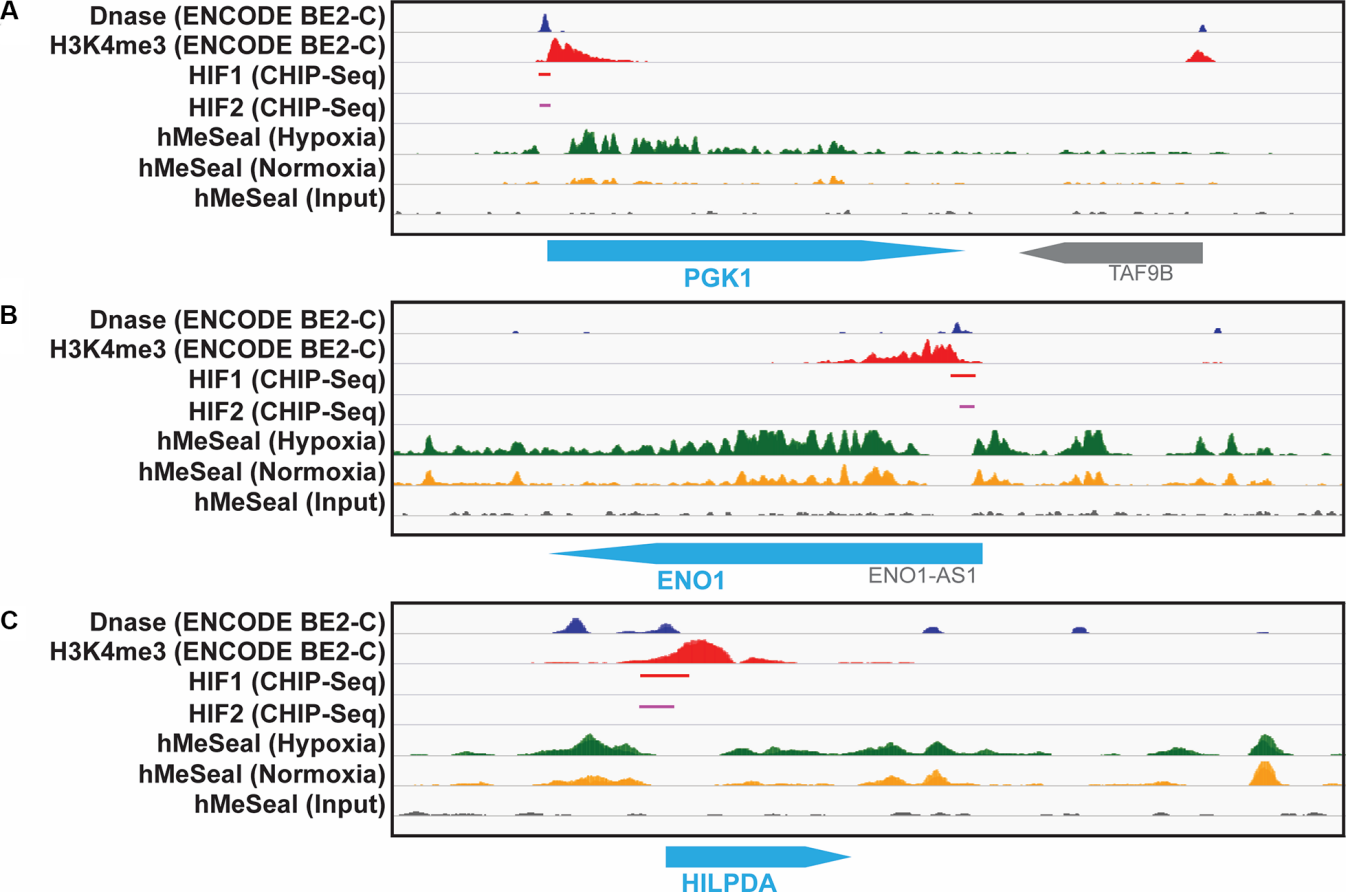
hMe-Seal and ENCODE data demonstrate an open chromatin structure and HIF binding at the promotor sites of identified genes Dnase-Seq (blue) and H3K4me3 (red) peaks are indicative of open chromatin regions. Chip-seq for HIF-1α (red) and HIF-2α (pink) show these transcription factors bind to these open promoter regions. hMe-Seal shows increased 5-hydroxymethylcytosine, another marker of open chromatin, in hypoxia (green) compared to normoxia (yellow) at each of these sites for the (**A**) PGK1, (**B**) ENO1, and (**C**) HILPDA genes.

In order to evaluate the link between hypoxia and our identified genes further, we analyzed hMe-Seal data from hypoxia exposed cells [18] and integrated several genomics datasets from ENCODE to test whether the candidate genes are HIF regulated. Our analysis demonstrated that five of these nine genes, *ENO1*, *PGK1*, *SLCO4A1*, *HK2*, and *HILPDA*, have increased 5-hydroxymethylcytosine levels in their regulatory regions in hypoxia compared to normoxia (Figure 3A–3C, Supplementary Table S8). Additionally, *ENO1*, *PGK1*, *HK2*, *MTFP1*, and *HILDPA* have putative *HIF-1α* and *HIF-2α* binding sites in their regulatory regions (Figure 3A–3C, Supplementary Figure S3A–S3F). Collectively these data suggest that many of our candidate genes have an open chromatin structure in hypoxia and are accessible to transcription factors including the hypoxia inducible factors. These observations provide additional support for the role of these genes in hypoxia response in neuroblastoma.

### Validation of hypoxia-regulated gene expression of the candidate genes in neuroblastoma cell lines

**Figure 4:**
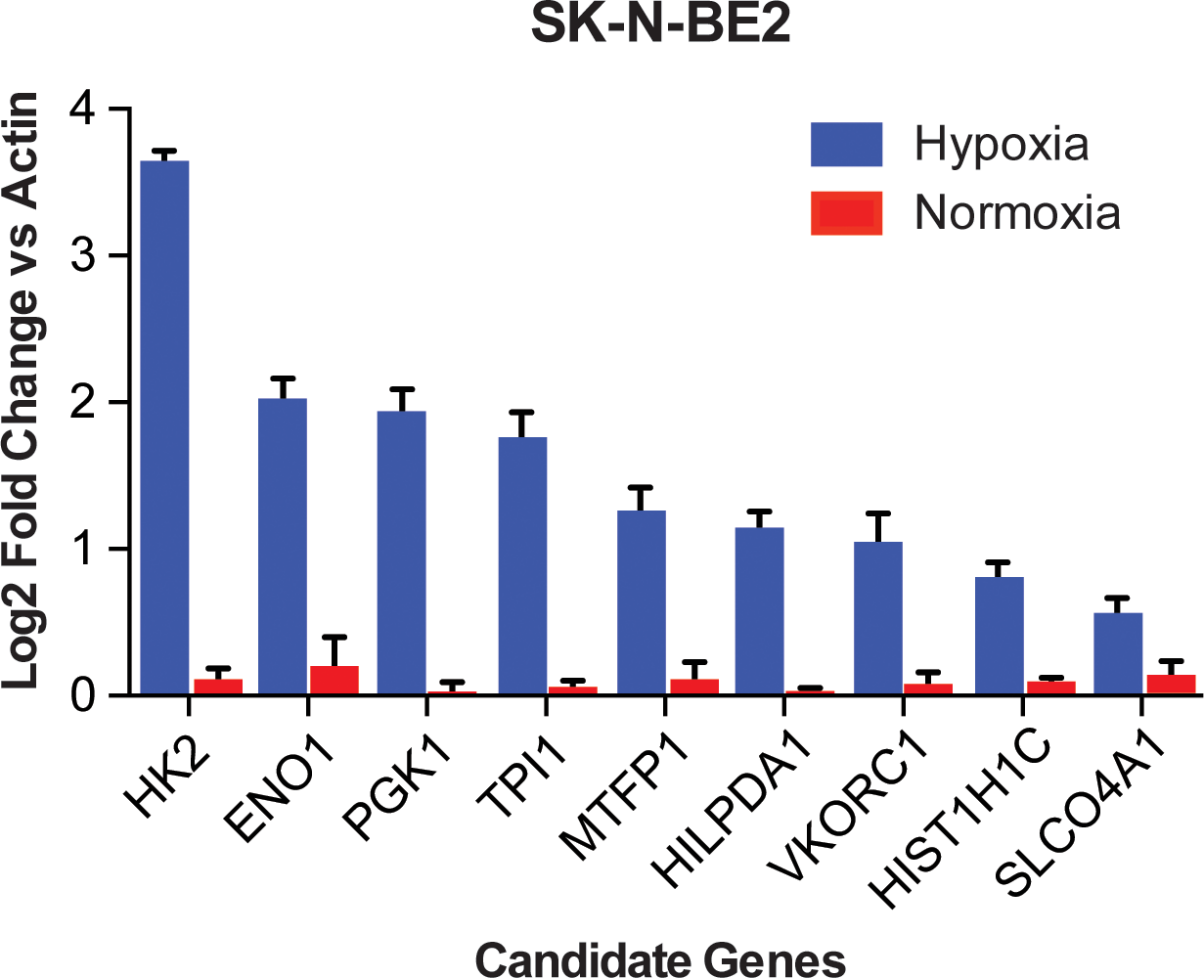
qRT-PCR shows regulation of each identified gene in neuroblastoma cell lines Log2 transformed expression values compared to actin for nine genes in hypoxia compared to normoxia in the SK-N-BE2 cell line. All genes were significantly up regulated in hypoxia (*P* < 0.05).

To verify that the candidate genes are differentially expressed in hypoxia, we performed qRT-PCR assays using cDNA isolated from the SK-N-BE2, SK-N-DZ, LAN-5, and LA1-55n cell lines. Of these cell lines, the SK-N-BE2 line was among the transcriptomic datasets that we analyzed initially, while the SK-N-DZ, LAN-5, and LA1-55n lines were not evaluated in the publically available expression data. Eight of the nine candidate genes were reliably up-regulated in hypoxia (*P* < 0.05) in each of the cell lines. This is consistent with up-regulation of these genes in hypoxia previously established in the dataset of 12 cell lines (Figure 4, Supplementary Figure S4A–S4C). More importantly, these genes were also upregulated in aggressive tumors in the two patient cohorts, suggesting the role of hypoxia in maintaining aggressive neuroblastoma. Additionally, *VKORC1* was significantly up-regulated in all cell lines except LAN-5 (*P* = 0.08), while *SLCO4A1* was only significantly elevated in SK-N-BE2 cells.

### Candidate genes are associated with poor prognosis

**Figure 5:**
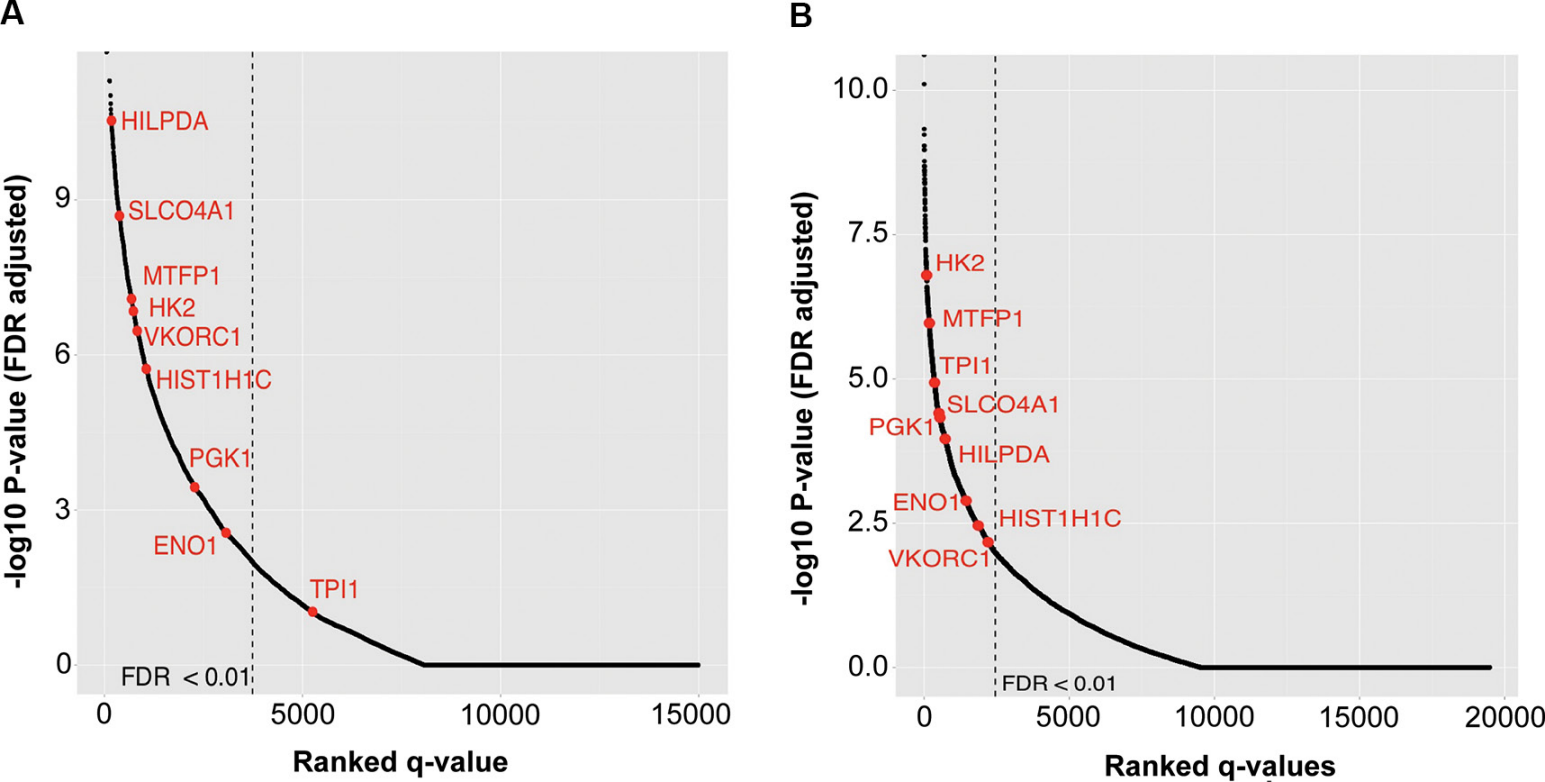
Kaplan-Meier analysis of all genes shows a significant association with outcome for eight of nine identified genes in both cohorts All genes on the array are ranked by most significant FDR of patients with high vs. low expression of each gene. Statistically significant genes are located to the left of the dashed lines. Our nine identified genes are in red. Rank of each identified gene for (**A**) Cohort 1 and (**B**) Cohort 2.

Having demonstrated that our identified genes are truly up-regulated in neuroblastoma cell lines, we sought to fully define their role in predicting poor patient outcomes. Higher expression of each candidate gene was also associated with patient survival as demonstrated by the Kaplan-Meier survival analysis in each patient cohort (Figure 5A and 5B, Supplementary Figure S5A and S5B), with the exception of the *TPI1* gene, which was significantly correlated with survival only in Cohort 2. We also performed a regression analysis for each gene to evaluate whether the changes in their expression are associated with poor prognosis. Consistent with the Kaplan-Meyer analyses, Cox regression analysis also showed that the expression differences in all of the nine candidate genes were associated with poor patient survival both in univariate and multivariate analysis controlling for *MYCN* status, stage, and age at diagnosis (Supplementary Table S9).

In order to verify the association between candidate genes and survival, we performed two additional analyses. First, we performed a permutation-based test to evaluate whether associations between DEGs and survival could be obtained by chance alone. To do so, we randomly selected eight DEGs from Cohort 1 and nine DEGs from Cohort 2, counted how many of these were associated with poor prognosis (FDR <1%) in both cohorts, and repeated this process 100,000 times. Our analyses showed that the associations between candidate genes and survival could not be observed by chance alone (*P* = 0.039 and *P* = 0.0087 for Cohorts I and 2, respectively). Second, we analyzed transcriptome data from a third independent cohort (Cohort 3), consisting of 233 neuroblastoma patients extracted from the larger group in E-MTAB-1781 who were not included in Patient Cohort 1 [19]. This cohort included patients with stage 1 (*n* = 41), stage 2 (*n* = 40), stage 3 (*n* = 22), stage 4 (*n* = 111), and stage 4S (*n* = 19) tumors. With the exception of *HIST1H1C* in Kaplan-Meier analysis, we again show that our identified genes are truly associated with poor outcome in neuroblastoma patients (Table 1).

**Table 1:** Validation of the association between nine identified genes and survival in patient Cohort 3 (n = 233)

Gene	Kaplan-Meier *q* value	Cox Regression Univariate *q* value	Cox Regression Multivariate *q* value
*HILPDA*	2.65E-03	1.69E-07	1.71E-04
*HK2*	2.65E-03	8.21E-08	5.95E-05
*SLCO4A1*	2.65E-03	1.59E-08	5.61E-06
*MTFP1*	9.56E-03	1.69E-07	1.98E-05
*PGK1*	1.94E-02	2.12E-03	2.18E-02
*ENO1*	1.94E-02	8.70E-03	1.93E-02
*VKORC1*	4.20E-02	1.59E-08	5.09E-04
*TPI1*	4.20E-02	2.45E-02	3.54E-02
*HIST1H1C*	2.70E-01	4.04E-06	2.07E-03

## DISCUSSION

In this study, we utilized diverse genomics data sources to identify a set of candidate genes computationally that appear to be both clinically and biologically important in neuroblastoma. By combining these diverse biologic datasets, we were able to confirm prior findings and demonstrate the clinical relevance of high expression of cell cycle genes. More importantly, using experimental data from neuroblastoma cell lines exposed to hypoxia, we were able to identify a small number of candidate genes that are not only important in hypoxia response but may also be important in patient survival.

Hypoxia has been shown to affect cell cycle and lead to aggressive phenotype in numerous types of cancer [20]. This effect is mediated by both HIF-1α and HIF-2α. Depending on the cancer type, high levels of one or both of these proteins has been associated with worse patient outcomes [21]. Additionally, the HIFs have also been shown to interact directly with the MYCN/MAX complex in neuroblastoma, which has a complex effect on cellular phenotype [22]. Previous work has shown that hypoxia signatures are correlated with outcome in patients with neuroblastoma [9, 23], but there have been few efforts to identify the subset of genes driving this finding.

Though further efforts are needed to confirm the full predictive potential of the genes we have identified, there are several lines of evidence to suggest that they are involved in aggressive disease and may guide efforts towards novel therapeutic approaches. Among the nine genes, seven were directly involved in metabolism and four, *PGK1*, *HK2*, *TPI1*, and *ENO1* are part of the glycolytic pathway and have previously been shown to be up-regulated in response to hypoxia, thus rendering them potential drug targets [24]. Glycolysis has been long associated with the cellular response to hypoxia and cellular proliferation [25]. In response to mitochondrial dysfunction and the loss of oxidative phosphorylation, HIF stabilization leads to up-regulation of the glycolytic pathway to allow for anaerobic metabolism. This effect is well documented in neuroblastoma cell lines [17]. Recent efforts have also demonstrated that high expression of *HK2*, one of our identified genes and the first enzymatic step in glycolysis, confers a malignant phenotype and chemotherapy resistance *in vitro*. Additionally, it was also shown that decreasing expression of *HK2* using shRNA both *in vitro* and *in vivo* slows the growth and metastatic potential of this tumor [26]. Furthermore, multiple drugs have been developed which inhibit *HK2* function and have been shown to be effective at decreasing tumor growth in preclinical testing [27]. These findings highlight the strength of our approach to go beyond expression signatures which have been difficult to implement in the clinic [28–30] and identify genes which may be functionally relevant. This emphasizes the need for further functional characterization of these genes specifically in the context of neuroblastoma, and potentially more generally in solid tumors as well.

We also identified five genes not in the glycolytic pathway which likely play an important role in the hypoxia response of neuroblastoma. *SLCO4A1* is a membrane transporter of which the only currently known solute is thyroid hormone. Interestingly, in lymphoblastoid cell lines, increased expression of this gene is associated with resistance to cisplatin, a backbone of neuroblastoma therapy (http://www.pacdb.org). *MTFP1* is a downstream target of *PI3KCA* and is involved in mitochondrial homeostasis [31]. *HILPDA* is a direct target of *HIF-1α* and *PPARα*, and elevated expression in hypoxia increases the number of lipid droplets in tumor cells [32]. Although *VKORC1* has been studied extensively in warfarin dosing [33], it has an unclear role in cancer biology. In hepatocellular carcinoma, *HIST1H1C* a gene involved in regulating higher order chromatin structures, may serve to maintain DNA methylation patterns.[34] Because our identified genes are part of the hypoxia response, and hypoxia is associated with aggressiveness in cancer [6, 35], these genes may play a role in increasing resistance to chemotherapy and/or promoting primary tumor growth and metastasis.

We analyzed a wide variety of transcriptomic and clinical data sets in order to identify genes of importance across 12 cell lines and over 500 patients for the biology and clinical phenotype of neuroblastoma. In doing so, we have elucidated a potential molecular mechanism driving hypoxia response in aggressive neuroblastoma. These results emphasize that neuroblastoma tumors rely on the glycolytic pathway for survival in the hypoxic conditions seen in patients and highlight a potential novel therapeutic strategy for this disease.

## MATERIALS AND METHODS

### Microarray expression data

Publically available microarray gene expression data from primary tumor samples and neuroblastoma cell lines were downloaded from GEO or EMBL. Patient Cohort 1 (*n* = 478) was characterized on the Agilent custom array (EMBL identification: E-MTAB-179) [11]. Patient Cohort 2 (*n* = 88) was characterized on the Affymetrix U133 Plus 2.0 Array (GEO identification: GSE16254) [13]. Patient Cohort 3 consisted of 233 neuroblastoma patients extracted from the larger group in E-MTAB-1781 who were not included in Patient Cohort 1. E-MTAB-1781 is an expansion of E-MTAB-179, and was generated using the same Agilent custom array. Eleven neuroblastoma cell lines (GI-LI-N, GI-ME-N, ACN, SHEP-2, SK-N-F1, SK-N-SH, SK-N-BE2c, IMR-32, LAN-1, SHEP-21N over-expressing *MYCN*, and SHEP-21N not over-expressing *MYCN*) grown in normoxia or 1% hypoxia were characterized on the Affymetrix U133 Plus 2.0 Array (Cell line experiment 2; GEO identification: GSE17714) [9]. Raw microarray expression data were normalized using the robust multi-array analysis algorithm to account for batch effects.[36] Normalized expression values of each probe were evaluated for DEGs between survivors and patients who died using linear models accounting for *MYCN* status as a fixed effect (y ~ status + MYCN). Differential expression in neuroblastoma cells lines grown in hypoxia or normoxia was evaluated using a generalized linear mixed effects model (GLMM) to account for the fixed effects of *MYCN* status, N or S type growth pattern [37], and specific cell line as the mixed effect (y ~ hypoxia + MYCN + (1|line) + growth pattern). Probes with the most significant *p*-value for each gene were selected for further analysis. FDRs were calculated from *p*-values by q-value method [38]. Genes with an FDR less than 0.01 and in the top or bottom 10% of log2 fold changes values between groups were considered significantly differentially expressed. It is a common practice to use log2 fold-change of 1.5 to identify differentially expressed genes. However, this threshold is arbitrary and several studies have shown that small changes in gene expression can be biologically relevant, especially in cancer [39]. An alternative approach is to use FDR, which can identify genes that consistently differ in expression between the two groups even if the magnitude of differences is small. By combining both, we are first identifying genes that are consistently differentially expressed between the two neuroblastoma groups and then selecting those genes that have changed with the largest magnitude. Due to the inherent heterogeneity of tumor samples, these thresholds were used to determine the candidate genes that are most likely to be biologically relevant in neuroblastoma.

### RNA-seq expression data

50 base pair, single stranded RNA-seq data were obtained from the SK-N-BE2 neuroblastoma cell line grown in 21% or 1% O_2_ (Cell line experiment 1; GEO accession GSE55391) [18]. Sequence quality was assessed with FASTQC v0.10.1 [40]. Reads were aligned to the hg19 reference genome with Tophat2 v2.0.13 [41]. Default settings in CuffDiff v2.2.1 [42] were used to identify DEGs in hypoxia compared to normoxia. Significance of differential expression between oxygen conditions was determined using thresholds identical to those used for microarray data.

### Survival analysis

Patients were categorized as having high or low expression of each gene using a sliding window to include at least two percent of patients. The log-rank test was used to calculate the *p*-value of the survival difference between high and low expression groups in each window. Multiple testing correction was done using the q-value method to account for both the number of genes in the dataset and the number of sliding windows per gene. The most significant q-value for each gene is reported. Cox proportional hazard models [43] were used to evaluate the effect of gene expression on overall survival. Both a univariate and a multivariate model accounting for *MYCN* status, stage, and age at diagnosis were utilized.

### Gene ontology enrichment, pathway analysis, and permutation testing

All GO pathway enrichment analyses were performed using the Lynx Platform [12]. Analysis was limited to the genes from each platform that passed quality control and were expressed. This included 14,386 genes from Patient Cohort 1, 16,219 genes from Patient Cohort 2, 14,688 genes from Cell line experiment 1, and 15,940 genes from Cell line experiment 2. A FDR of less than 5% was used to determine statistical significance. In order to determine if the number of identical DEG in each set was greater than would be expected by chance, we performed permutation testing. For each dataset in an analysis, genes were randomly selected corresponding to the number of DEGs identified. We then counted the number of genes common in each randomly generated gene set and repeated the procedure 100,000 times per analysis. *P*-values were calculated by dividing the number of occurrences of randomly overlapping genes equal or greater to the number of genes in our experimentally derived lists by 100,000.

### Chromatin structure and HIF-binding

hMe-Seal experiments to quantify a marker of open chromatin 5-hydroxymethylcytosine, from two biologic replicates of SK-N-BE2 cells grown in normoxia or hypoxia were conducted as previously described [18]. 5-hydroxymethylcytosine peaks were quantified from BAM files using MACS2 v2.1.0 using default settings [44]. Diffbind [45] was used to determine both the *p*-values and FDR of called peaks between hypoxic and normoxic conditions. An FDR of less than 5% was considered significant.

### Cell culture

Neuroblastoma cell lines SK-N-BE2, SK-N-DZ, LAN-5, and LA1-55n were maintained in RPMI 1640 media (ThermoFisher Scientific) supplemented with 10% heat-inactivated fetal bovine serum. SK-N-DZ was purchased from ATCC. All cell lines were authenticated within six months of all experiments by short tandem repeat (STR) profiling and profiles were found to be identical to known profiles for the cell lines. All cell lines tested negative for Mycoplasma contamination using the MycoAlert detection assay (Lonza). Cells were seeded 18–24 hours prior to hypoxia exposure. For hypoxic conditions, cells were incubated in 1% O_2_ and 5% CO_2_. For normoxic conditions cells were incubated at 21% O_2_ and 5% CO_2_. All experiments were performed in triplicate.

### RNA extraction and qRT-PCR

RNA was isolated from cell lines using Trizol reagent (ThermoFisher Scientific). First-strand cDNA synthesis was performed using the SuperScript III First-Strand Synthesis System (ThermoFisher Scientific). Gene-specific primers were obtained for each gene (Integrated DNA Technologies Coralville, IA) and were normalized to the housekeeping gene *beta-Actin* (Supplementary Table S1). The real-time quantitative PCR was conducted using the 7500 Fast Real-Time PCR System (ThermoFisher Scientific) according to manufacturer's protocol. The calculation of the gene expression levels followed the 2^−ΔΔCT^ rule.

### Statistics

All statistical analyses were performed in R version 3.2.1.

## SUPPLEMENTARY MATERIALS FIGURES AND TABLES




