# Supplementary material for: Integrative genomics reveals hypoxia inducible genes that are associated with a poor prognosis in neuroblastoma patients
Source: Oncotarget. 2016 Oct 17;7(47):76816–26. doi: 10.18632/oncotarget.12713 (PMC5340231; doi:10.18632/oncotarget.12713)
Supplement: Supplementary file 1 [file oncotarget-07-76816-s001.pdf]

Integrative genomics reveals hypoxia inducible genes that are associated with a poor prognosis in neuroblastoma patients

Supplementary Materials

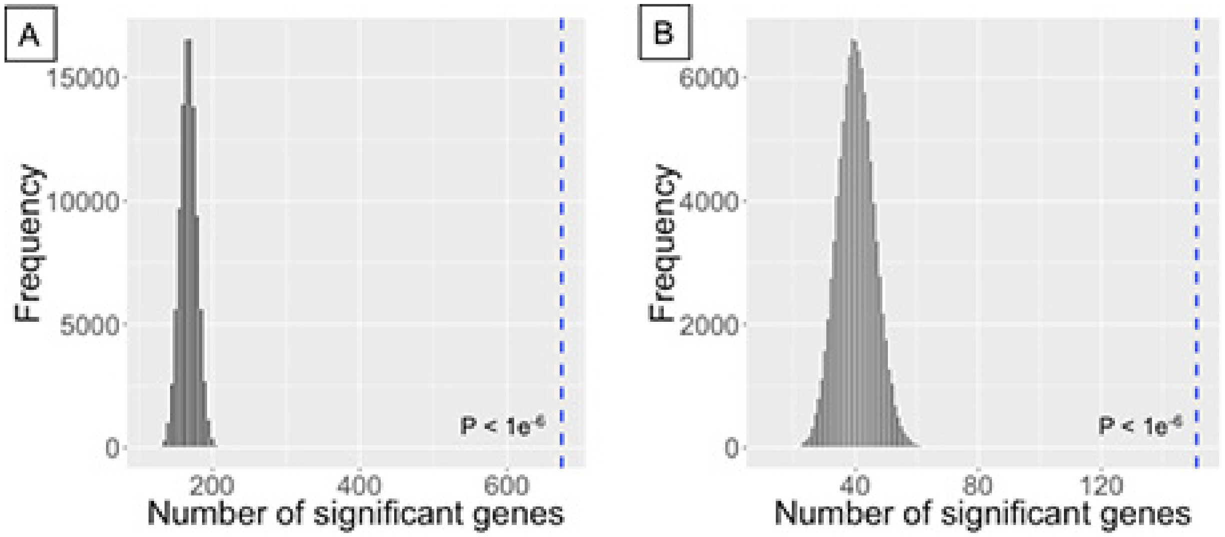

Supplementary Figure S1: The number of DEGs is greater than would be expected by chance. Histograms of 100,000 random samplings is shown for (**A**) patient cohorts and (**B**) cell line experiments. The dashed line indicates the number of common DEGs identified in each analysis.

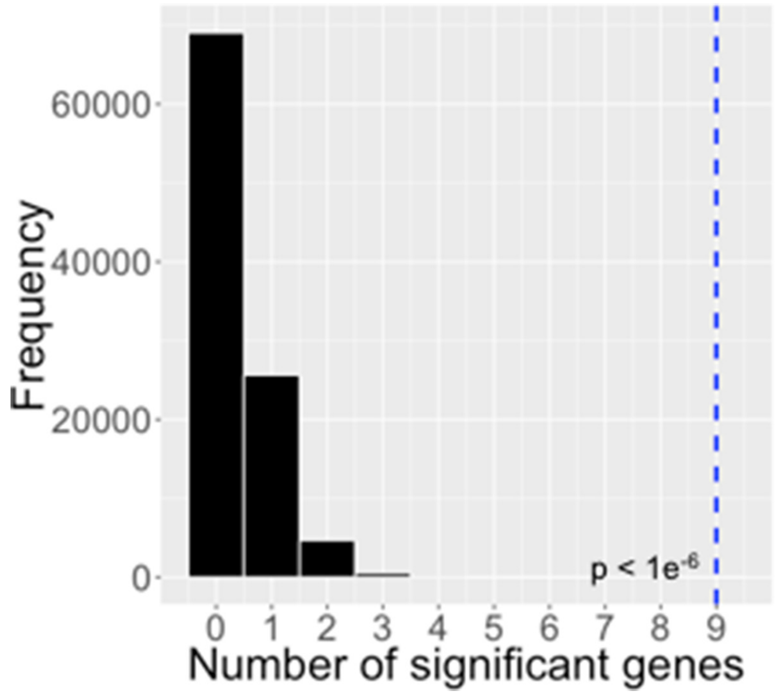

Supplementary Figure S2: The number of DEGs of consistent directionality in all four cohorts is greater than would be expected by chance. A histogram of 100,000 random samplings for each of the patient cohorts and cell line experiments is shown. The dashed line indicates the number of common DEGs identified.

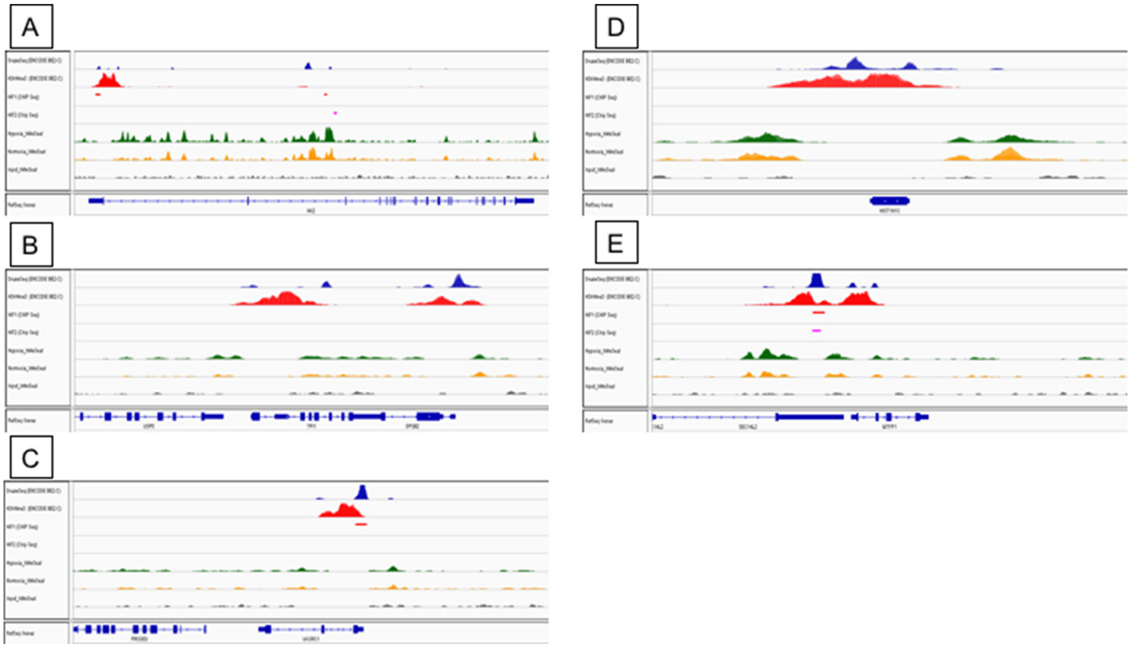

Supplementary Figure S3: hMe-Seal and ENCODE data demonstrate an open chromatin structure and HIF binding at the promotor sites of identified genes. DnaseSeq (blue) and H3K4me3 (red) peaks are indicative of open chromatin regions. Chip-Seq for *HIF-1α* (red) and *HIF-2α* (pink) show these transcription factors bind to some of these open promoter regions. hMe-Seal shows 5-hydroxymethylation, another marker of open chromatin, in hypoxia (green) and normoxia (yellow) at each of these sites for the (**A**) *HK2*, (**B**) *SLCO4A1*, and (**C**) *TPI1*, (**D**) *VKORC1*, (**E**) *HIST1H1C*, and (**F**) *MTFP1* genes.

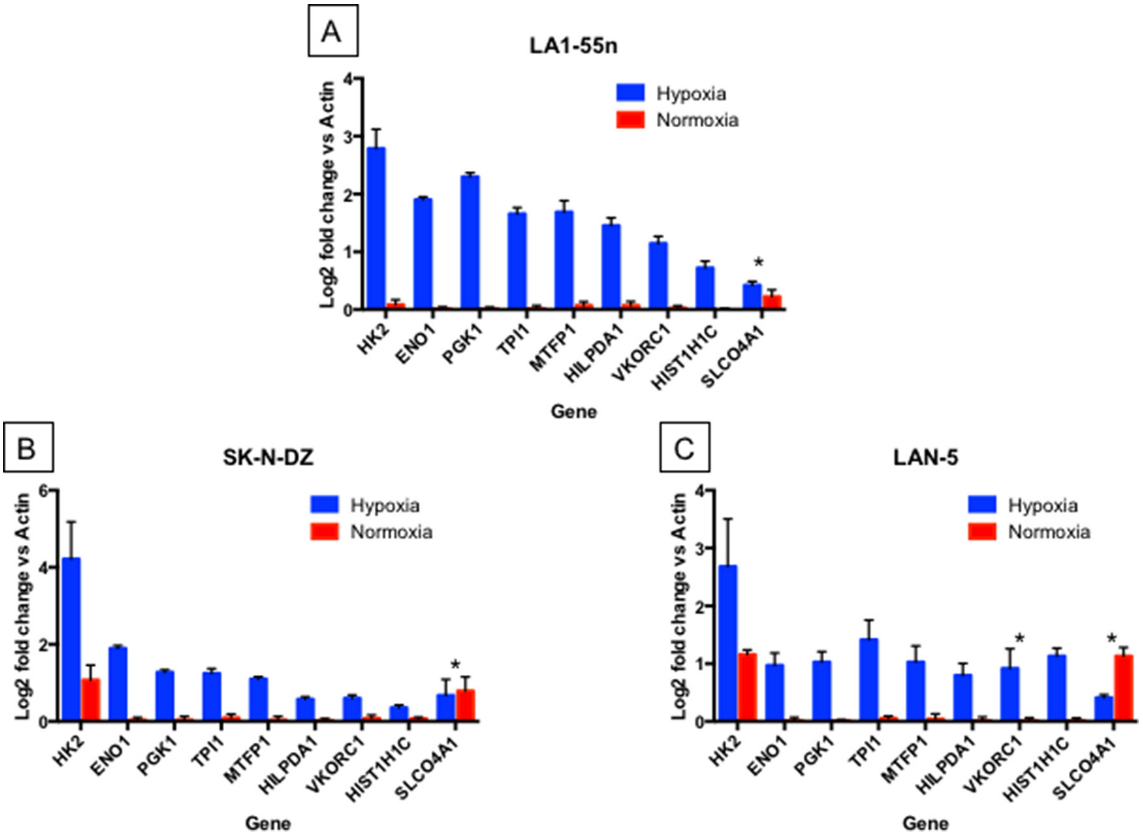

Supplementary Figure S4: qRT-PCR validation of nine genes in the (A) LA1-55n, (B) SK-N-DZ, and (C) LAN-5 neuroblastoma cell lines. All genes have increased expression in hypoxia ($P < 0.05$) except as noted with an *.

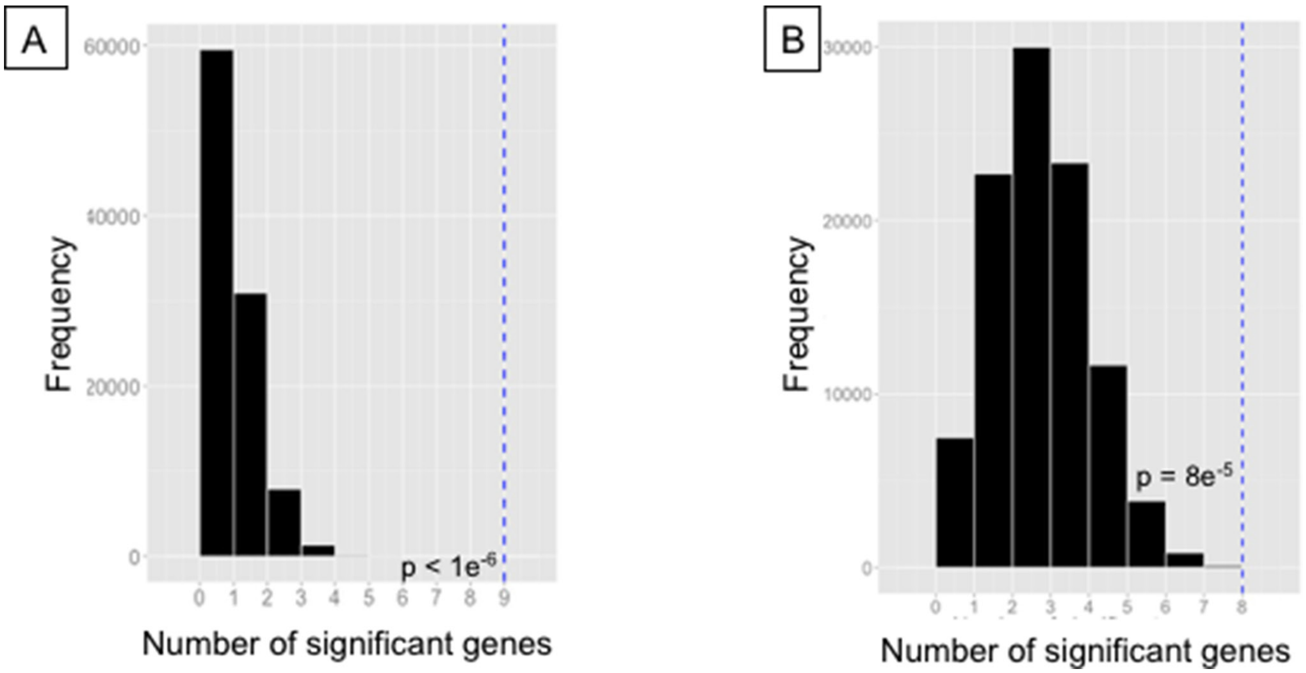

Supplementary Figure S5: The number of identified genes significantly associated with survival by log-rank test is greater than would be expected by chance. Histograms of 100,000 random samplings for each of the patient cohorts is shown. The dashed line indicates the number of genes with an FDR < 0.01 in each analysis.

Supplementary Table S1: Primers used for qPCR analysis

GeneName	Amplicon Length	Forward_Primer	Forward_ Primer_Tm	Reverse_Primer	Reverse_ Primer_Tm	Primer_ SourceID
ACTIN-b	NA	TGGCACCACAC CTTCTACAA	NA	CCAGAGGCGTAC AGGGATAG	NA	Mike Bolt
PDK1	105	ATTTTCCTCAAA GGAACGCC	60	CAACAGAGGTGTT TACCCCC	59	PrimerDepot
HIST1H1C	98	AGCCTTAGCAGC ACTTTTGG	60	ACACCGAAGAAAG CGAAGAA	60	PrimerDepot
MTFP1	95	TTCCCCACTGTTG GGTAGAG	60	CTGTTGACCATCCCC ATCAT	61	PrimerDepot
VKORC1	102	CTCAGCAGCATCAG GACAGA	60	CCTCAATCAATCCAA CAGCA	60	PrimerDepot
HK2	110	AGCCCTTTCTCCAT CTCCTT	59.28	AACCATGACCAAG TGCAGAA	59.139	PrimerDepot
ENO1	80	TGTTCCTTCCAGG TGTCTC	59	CGTTCAGTTTCTTG CTAACCA	59	http:// updepla1srv1. epfl.ch/getprime/
SLCO4A1	94	GGGAGTGGCCAAG TGGTATC	61.684	GAGGCCAGAGCG TGGAG	60.682	PrimerDepot
HILPDA	139	GCAGAGAAACAGAG CTGCCT	59.898	CTTCTGCGCTGGTG CTTAGT	60.73	PrimerDepot
TPI1	121	ATGGCTGAAGTCCA ACGTCT	60	AAGGAAGCCATCCA CATCAG	60	http://medgen. ugent.be/ rtprimerdb/ assay_report. php?assay_ id=1082

Supplementary Table S2: Gene Ontology analysis of up-regulated differentially expressed genes in patient cohort 1. See Supplementary_Table_S2

Supplementary Table S3: Gene Ontology analysis of up-regulated differentially expressed genes in patient cohort 2. See Supplementary_Table_S3

Supplementary Table S4: Gene Ontology analysis of up-regulated differentially expressed genes in both patient cohorts. See Supplementary_Table_S4

Supplementary Table S5: Gene Ontology analysis of up-regulated differentially expressed genes in the SK-N-BE2 cell line. See Supplementary_Table_S5

Supplementary Table 6: Gene Ontology analysis of up-regulated differentially expressed genes in 11 neuroblastoma cell lines

Feature ID	Name	Data Source	In Query	In Test Set	Genes	FDR
GO:0061621	canonical glycolysis	UniProt-GOA	13	27	ALDOA ALDOC ENO1 ENO2 GPI HK1 HK2 PFKFB4 PFKP PGAM1 PGK1 PKM TPI1	5.42E-09
GO:0006006	glucose metabolic process	UniProt-GOA	20	111	ALDOA ALDOC ENO1 ENO2 GBE1 GPI GYS1 HK1 HK2 PDK1 PDK3 PFKFB4 PFKP PGAM1 PGK1 PGM1 PGM2L1 PKM SORD TPI1	8.76E-09
GO:0006096	glycolytic process	UniProt-GOA	12	31	ALDOA ALDOC ENO1 ENO2 GPI HK1 HK2 PFKP PGAM1 PGM1 PKM TPI1	1.11E-07
GO:0001666	response to hypoxia	UniProt-GOA	20	144	ADM ALDOC ALKBH5 ANG APOLD1 BNIP3 CITED2 CXCR4 DDIT4 EGLN1 EGLN3 ITPR1 LOXL2 NOL3 P2RX3 PLOD1 PLOD2 PPARA RAF1 VEGFA	2.97E-07
GO:0005975	carbohydrate metabolic process	UniProt-GOA	23	312	ALDOA ALDOC ENO1 ENO2 GBE1 GPI GUSBP11 GYS1 HK1 HK2 MPI PFKFB4 PFKP PGAM1 PGK1 PGM1 PKM SDC3 SLC2A1 SLC2A3 SORD ST3GAL3 TPI1	0.001
GO:0006094	gluconeogenesis	UniProt-GOA	9	42	ALDOA ALDOC ENO1 ENO2 GPI PGAM1 PGK1 PGM1 TPI1	0.001
GO:0018401	peptidyl-proline hydroxylation to 4-hydroxy-L-proline	UniProt-GOA	4	5	EGLN1 EGLN3 P4HA1 P4HA2	0.03

Supplementary Table S7: Gene Ontology analysis of up-regulated, differentially expressed genes in both cell line datasets

Feature ID	Name	Data Source	In Query	In Test Set	Genes	FDR
GO:0061621	canonical glycolysis	UniProt-GOA	12	24	ALDOA ALDOC ENO1 ENO2 GPI HK1 HK2 PFKFB4 PFKP PGAM1 PGK1 TPI1	1.85E-13
GO:0006006	glucose metabolic process	UniProt-GOA	16	102	ALDOA ALDOC ENO1 ENO2 GBE1 GPI HK1 HK2 PDK1 PDK3 PFKFB4 PFKP PGAM1 PGK1 PGM1 TPI1	2.46E-12
GO:0006096	glycolytic process	UniProt-GOA	11	25	ALDOA ALDOC ENO1 ENO2 GPI HK1 HK2 PFKP PGAM1 PGM1 TPI1	4.13E-12
GO:0001666	response to hypoxia	UniProt-GOA	13	124	ADM ALDOC ALKBH5 BNIP3 CXCR4 DDIT4 EGLN1 EGLN3 LOXL2 P2RX3 PLOD1 PLOD2 VEGFA	7.27E-08
GO:0006094	gluconeogenesis	UniProt-GOA	9	37	ALDOA ALDOC ENO1 ENO2 GPI PGAM1 PGK1 PGM1 TPI1	8.05E-08
GO:0005975	carbohydrate metabolic process	UniProt-GOA	17	281	ALDOA ALDOC ENO1 ENO2 GBE1 GPI HK1 HK2 MPI PFKFB4 PFKP PGAM1 PGK1 PGM1 SLC2A1 SLC2A3 TPI1	1.55E-07
GO:0055114	oxidation-reduction process	UniProt-GOA	18	440	BCKDHA EGLN1 EGLN3 KCNAB2 KDM3A KDM4B KDM5B KDM6B LOX LOXL2 MAOA P4HA1 P4HA2 PAM PLOD1 PLOD2 SCD VKORC1	1.40E-05
GO:0071456	cellular response to hypoxia	UniProt-GOA	9	85	BNIP3 CA9 EGLN1 EGLN3 FAM162A NDRG1 RORA STC1 VEGFA	3.55E-05
GO:0018401	peptidyl-proline hydroxylation to 4-hydroxy-L-proline	UniProt-GOA	4	6	EGLN1 EGLN3 P4HA1 P4HA2	4.56E-04
GO:0044281	small molecule metabolic process	UniProt-GOA	25	1123	ALDOA ALDOC AMPD3 BCKDHA CA12 CA9 ENO1 ENO2 GBE1 GPI HK1 HK2 INSIG2 MAOA PDK1 PDK3 PFKFB4 PFKP PGAM1 PGK1 PGM1 SCARB1 SLC2A1 SLC2A3 TPI1	0.001
GO:0046835	carbohydrate phosphorylation	UniProt-GOA	4	22	HK1 HK2 PFKFB4 PFKP	0.024
GO:0051156	glucose 6-phosphate metabolic process	UniProt-GOA	3	7	GPI HK1 HK2	0.025
GO:0030199	collagen fibril organization	UniProt-GOA	4	26	COL5A1 LOX LOXL2 P4HA1	0.036
GO:0061418	regulation of transcription from RNA polymerase II promoter in response to hypoxia	UniProt-GOA	4	27	CA9 EGLN1 EGLN3 VEGFA	0.039

Supplementary Table S8: 5-hydroxymethylation as a measure of open chromatin is increased in hypoxia for six of nine identified genes

Gene	Chromosome	Start	End	5-hMc Fold change	FDR
ENO1	chr1	8.93E+06	8.94E+06	1.57	3.39E-46
PGK1	chrX	7.74E+07	7.74E+07	2.01	1.22E-24
SLCO4A1	chr20	6.14E+07	6.15E+07	2.39	3.13E-26
HK2	chr2	7.51E+07	7.51E+07	1.88	2.22E-12
MTFP1	chr22	3.08E+07	3.08E+07	1.01	0.000247
HILPDA	chr7	1.28E+08	1.28E+08	1.25	0.0145

Supplementary Table S9: Cox regression shows significant association between gene expression and survival for both patient cohorts in both univariate and multivariate analysis

Gene	Cohort 1			Cohort 2		
	Fold change	Univariate *q* value	Multivariate *q* value	Fold change	Univariate *q* value	Multivariate *q* value
HIST1H1C	1.69	1.11E-17	2.42E-09	1.79	1.97E-02	1.25E-02
HILPDA	1.26	1.11E-17	1.92E-08	1.61	7.06E-03	4.40E-03
HK2	1.63	1.11E-17	1.32E-06	3.22	1.90E-04	1.17E-04
MTFP1	1.25	1.11E-17	4.18E-06	1.43	4.03E-03	2.51E-03
SLCO4A1	1.19	1.11E-17	4.89E-06	1.48	2.85E-02	1.81E-02
VKORC1	1.18	1.11E-17	1.99E-05	1.31	2.57E-02	3.84E-01
PGK1	1.20	8.66E-15	1.88E-04	1.41	1.78E-04	1.10E-04
ENO1	1.22	1.67E-11	3.90E-04	1.45	6.09E-04	3.80E-04
TPI1	1.13	1.38E-05	4.63E-03	1.38	1.08E-03	6.68E-04
